# Regulation of the β-hemolysin gene cluster of *Streptococcus anginosus* by CcpA

**DOI:** 10.1038/s41598-018-27334-z

**Published:** 2018-06-13

**Authors:** Richard Bauer, Stefanie Mauerer, Barbara Spellerberg

**Affiliations:** Institute of Medical Microbiology and Hospital Hygiene, University of Ulm, Ulm, Germany

## Abstract

*Streptococcus anginosus* is increasingly recognized as an opportunistic pathogen. However, our knowledge about virulence determinants in this species is scarce. One exception is the streptolysin-S (SLS) homologue responsible for the β-hemolytic phenotype of the *S*. *anginosus* type strain. In *S*. *anginosus* the expression of the hemolysin is reduced in the presence of high glucose concentrations. To investigate the genetic mechanism of the hemolysin repression we created an isogenic *ccpA* deletion strain. In contrast to the wild type strain, this mutant exhibits hemolytic activity in presence of up to 25 mM glucose supplementation, a phenotype that could be reverted by *ccpA* complementation. To further demonstrate that CcpA directly regulates the hemolysin expression, we performed an *in silico* analysis of the promoter of the SLS gene cluster and we verified the binding of CcpA to the promoter by electrophoretic mobility shift assays. This allowed us to define the CcpA binding site in the SLS promoter region of *S*. *anginosus*. In conclusion, we report for the first time the characterization of a potential virulence regulator in *S*. *anginosus*.

## Introduction

Bacteria of the *Streptococcus anginosus* group (SAG) are considered commensals of mucosal membranes^1^ but an increasing number of reports in recent years demonstrate their clinical importance^2–5^. In a large population based investigation the incidence rate of invasive SAG infections (8.65/100 000 population) even exceeds the combined incidence rate of group A and B streptococcal invasive infections (7.40/100 000 population)^6^. Bacteria of the SAG (*Streptococcus anginosus*, *Streptococcus constellatus* and *Streptococcus intermedius*) can frequently be isolated from abscesses and blood samples^5,7^ and are associated with different clinical pictures^1,7,8^. The knowledge about the virulence gene repertoire of these species is rare and mainly relies on interpretation of genome data^9^. One exception is the gene locus responsible for the β-hemolytic phenotype of the *S*. *anginosus* type strain which displays high homologies to streptolysin-S (SLS) of *Streptococcus pyogenes*^10,11^. SLS of *S*. *pyogenes* is considered a major virulence factor that is cytolytic for a variety of eukaryotic cells and has been shown to play an important role in *in vivo* models of skin and soft tissue infections^12^. The SLS molecule is a posttranslationally modified peptide of 2.7 kDa that is present in numerous pathogenic streptococci and other Gram-positive pathogens such as clostridia and listeria. It belongs to the TOMM (thiazole/oxazole-modified microcins) family of virulence peptides^13^. SLS of *S*. *anginosus* is able to lyse erythrocytes of different origins including human, sheep and chicken and it is inhibited in the presence of high glucose levels in the growth medium^14^.

Bacteria tightly regulate the uptake and consumption of different carbohydrates as the simultaneous utilization of all accessible sugars would be energetically inefficient. This regulatory process leading to a hierarchical metabolism of sugars is called carbon catabolite repression (CCR)^15^. In Gram-positive bacteria the catabolite control protein A (CcpA) is the major player in CCR^16^ although CcpA independent CCR mechanisms are well documented^17–19^. In order to be able to bind to DNA, CcpA needs to be activated by a phosphorylated form (serine-46) of the histidine-containing phosphocarrier protein (HPr). The availability of glucose and other preferred sugars leads to increased fructose-1,6-bisphosphate (FBP) levels in the cell. Accumulation of FBP activates the kinase activity of the HPr kinase/phosphorylase which phosphorylates the serine-46 residue of HPr. Thus high FBP levels indirectly activate CcpA via HPr^20^. Activated CcpA binds to catabolite responsive elements (*cre*) located in the promoter region of target genes predominantly resulting in the downregulation of gene expression. Studies in *Bacillus subtilis* and other Gram-positive bacteria have determined the consensus sequence of the *cre* sites which consist of highly degenerate pseudo-palindromes^21–24^. It was demonstrated that CCR is one of the most important regulatory processes in different bacteria with 5–10% of genes affected by CCR^23,25,26^. CcpA thereby mainly represses the expression of genes involved in consumption of alternative sugars and it activates expression of genes needed for glucose metabolism^27^. However, CcpA was also demonstrated to regulate virulence gene expression in different bacterial pathogens and thus represents an important link between virulence and metabolism^24,28–31^. In *S*. *pyogenes* CcpA controls SLS expression by binding to a *cre* site in the bacterial promoter^24^.

Since the *S*. *anginosus* SLS genes are repressed by high glucose levels^14^, we investigated if CcpA is responsible for this effect. Through the generation of various deletion mutants and EMSA assays, we show that SLS expression in this species is under control of the CcpA regulator and we identified the CcpA binding site in the SLS promoter region of *S*. *anginosus*. Thus we are able to demonstrate for the first time the regulation of a putative virulence gene in the emerging pathogen *S*. *anginosus*.

## Results

### CcpA affects SLS expression

**Figure 1:**
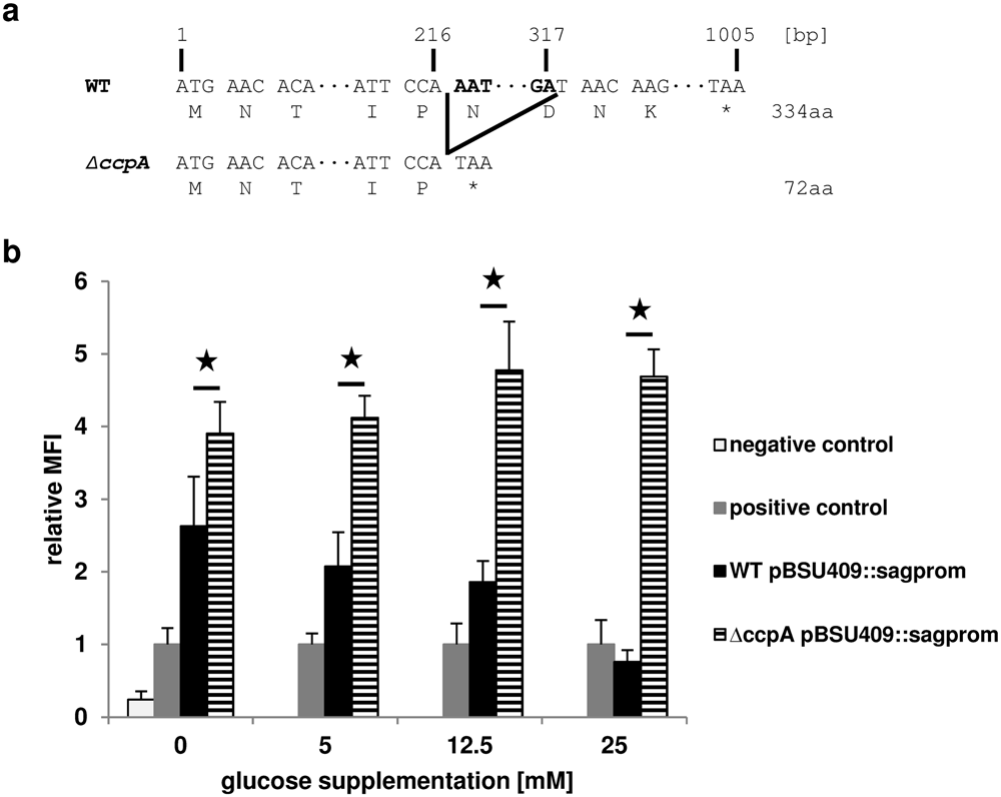
(**a**) Schematic representation of the 100 bp deletion in the *S*. *anginosus ∆ccpA* strain. The site of deletion is marked with black lines and the deleted base pairs in bold. The 100 bp deletion results in a frameshift and a premature stop codon (*). (**b**) Effect of glucose supplementation on SLS promoter activity. The activity of the SLS promoter was determined using an EGFP reporter plasmid. The relative mean fluorescence intensity (MFI) of cells grown in THY medium supplemented with the indicated glucose concentrations is shown in comparison to the positive control. Negative control: *S*. *anginosus* pBSU409; positive control: *S*. *anginosus* pBSU409*::cfbprom*; WT: *S*. *anginosus* type strain; ΔccpA: *S*. *anginosus ΔccpA* strain. The mean values and standard deviations of five independent experiments are shown. Mann-Whitney-U test was performed to illustrate significant difference to *S*. *anginosus* pBSU409*::sagprom* (p < 0.05).

In previous experiments we noticed that the SLS expression of *S*. *anginosus* was reduced with increasing glucose concentrations in the growth medium^14^. To elucidate the molecular mechanism behind this phenomenon and the potential role of CcpA on reducing the SLS expression, a *S*. *anginosus ΔccpA* strain was constructed (Fig. 1a). It carries a 100 bp deletion resulting in a frameshift and a truncated CcpA protein. This strain was transformed with the promoter reporter plasmid pBSU409*::sagprom*^11^ that contains the EGFP under the control of the SLS promoter^10^. To determine the influence of sugar on SLS expression in a *ccpA* negative background, the activity of the promoter was quantified at a range of different glucose concentrations in the medium (Fig. 1b). The dose-dependent reduction of SLS expression observed in the *S*. *anginosus* type strain is absent in the *S*. *anginosus ΔccpA* strain that showed a constant relative fluorescence over all tested glucose concentrations.

### Effect of *ccpA* knockout on SLS activity

**Figure 2:**
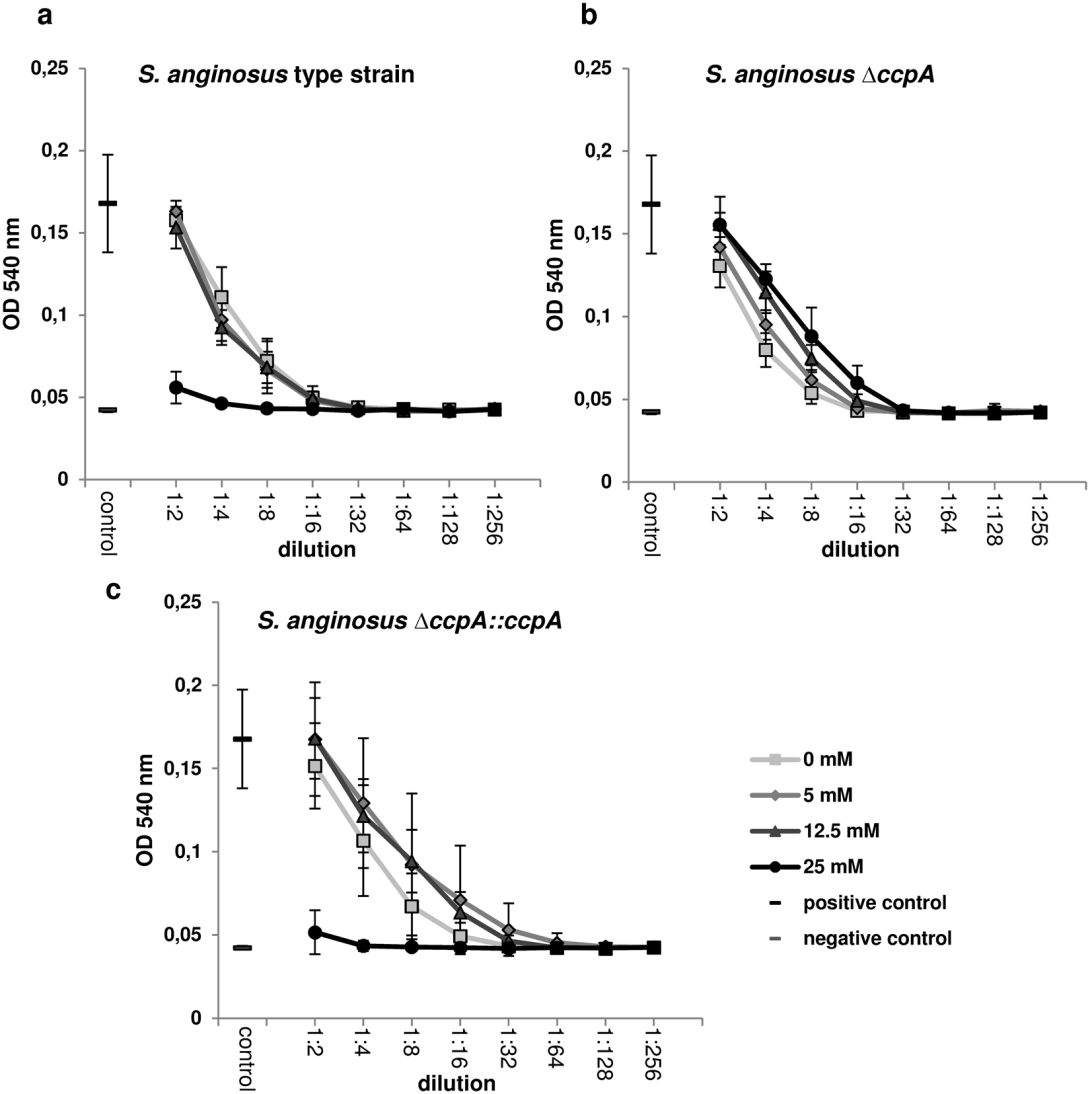
Hemolytic activity of *S*. *anginosus* strains for human erythrocytes. Bacteria were grown in THY medium supplemented with the indicated glucose concentrations. The hemolytic behavior of the *S*. *anginosus* type strain (**a**), the *S*. *anginosus ΔccpA* strain (**b**) and the complemented *S*. *anginosus ΔccpA::ccpA* strain (**c**) is illustrated for different bacterial cell dilutions. Positive control: ddH_2_O; negative control: assay buffer. The mean values and standard deviations of five independent experiments are shown.

To investigate the effect of the reduced SLS expression on the hemolysin activity, a functional hemolytic assay with human erythrocytes was performed with cells growing in the presence of different glucose concentrations (Fig. 2). The *S*. *anginosus* type strain incubated in medium supplemented with up to 12.5 mM glucose showed regular β-hemolysis, while cells grown in medium with the supplementation of 25 mM glucose were non-hemolytic. In contrast to the wild type, the *ΔccpA* strain was hemolytic in the presence of all tested glucose concentrations. To verify that the *ccpA* deletion is responsible for this phenotypic difference a *ccpA* complementation strain was created that contains the *ccpA* gene, carrying a silent mutation, in the original locus followed by an erythromycin resistance cassette. This *S*. *anginosus ΔccpA::ccpA* strain exhibits the same hemolytic phenotype as the wild type strain, showing a lack of hemolysis for cells that were grown in medium supplemented with 25 mM of glucose.

### *In silico* prediction of CcpA binding sites

**Figure 3:**
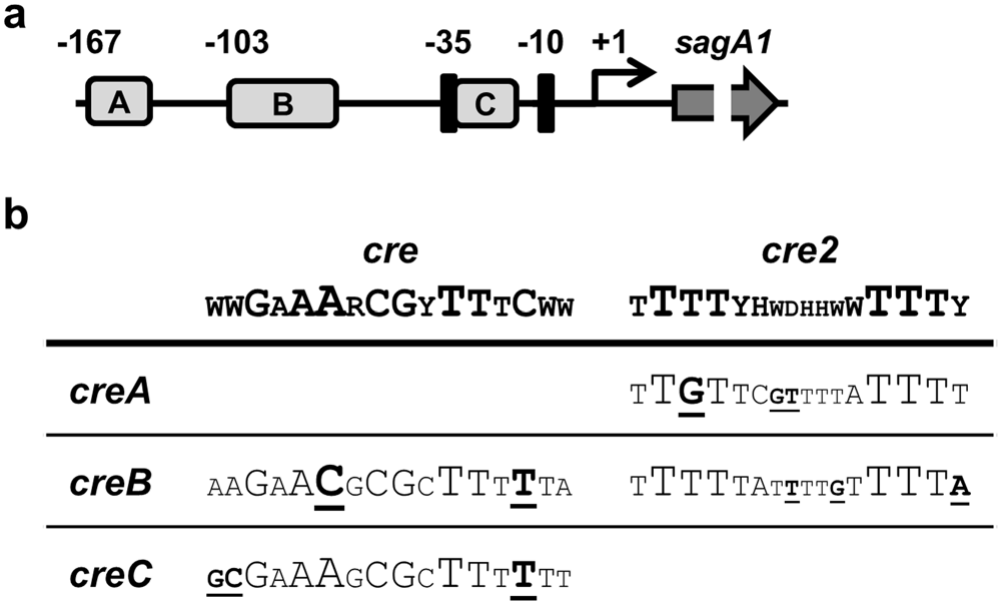
(**a**) The locations of the putative *cre* sites are illustrated in respect to the transcription start site (arrow). A: *creA*, B: *creB*, C: *creC*. (**b**) Comparison of the *in silico* predicted *cre* site in the SLS promoter and the published consensus *cre* sites. The size of the single nucleotides corresponds to their conservation. The *creB* site consists of a *cre* homologue overlapping a potential *cre2* site. Mismatches to the consensus sequences are underlined and bold.

CcpA is an extensively investigated regulator in Gram-positive bacteria and consensus sequences of its DNA binding site, the catabolite responsive element (*cre*), have been determined (http://regprecise.lbl.gov/RegPrecise/collection_tf.jsp?collection_id = 163). Recently a second CcpA binding site (*cre2*) was identified in *Streptococcus suis*^23^. To substantiate the involvement of CcpA in the control of SLS, we screened the SLS promoter for the presence of potential *cre* sites allowing three mismatches to the consensus sequences. This led to the identification of three putative CcpA binding sites in the SLS promoter of the *S*. *anginosus* type strain (Fig. 3a). One of these *cre* sites (*creA*) is located 167 bp upstream of the transcription start site and shows homologies to the *cre2* site. The *creB* sequence consists of a *cre* homologue overlapping a potential *cre2* site and the third sequence (*creC*) is located in between the −35 and −10 region of the SLS promoter. It contains three mismatches to less conserved nucleotides of the consensus *cre* site (Fig. 3b).

### *CreC* site mutation abolishes glucose dependent SLS repression

**Figure 4:**
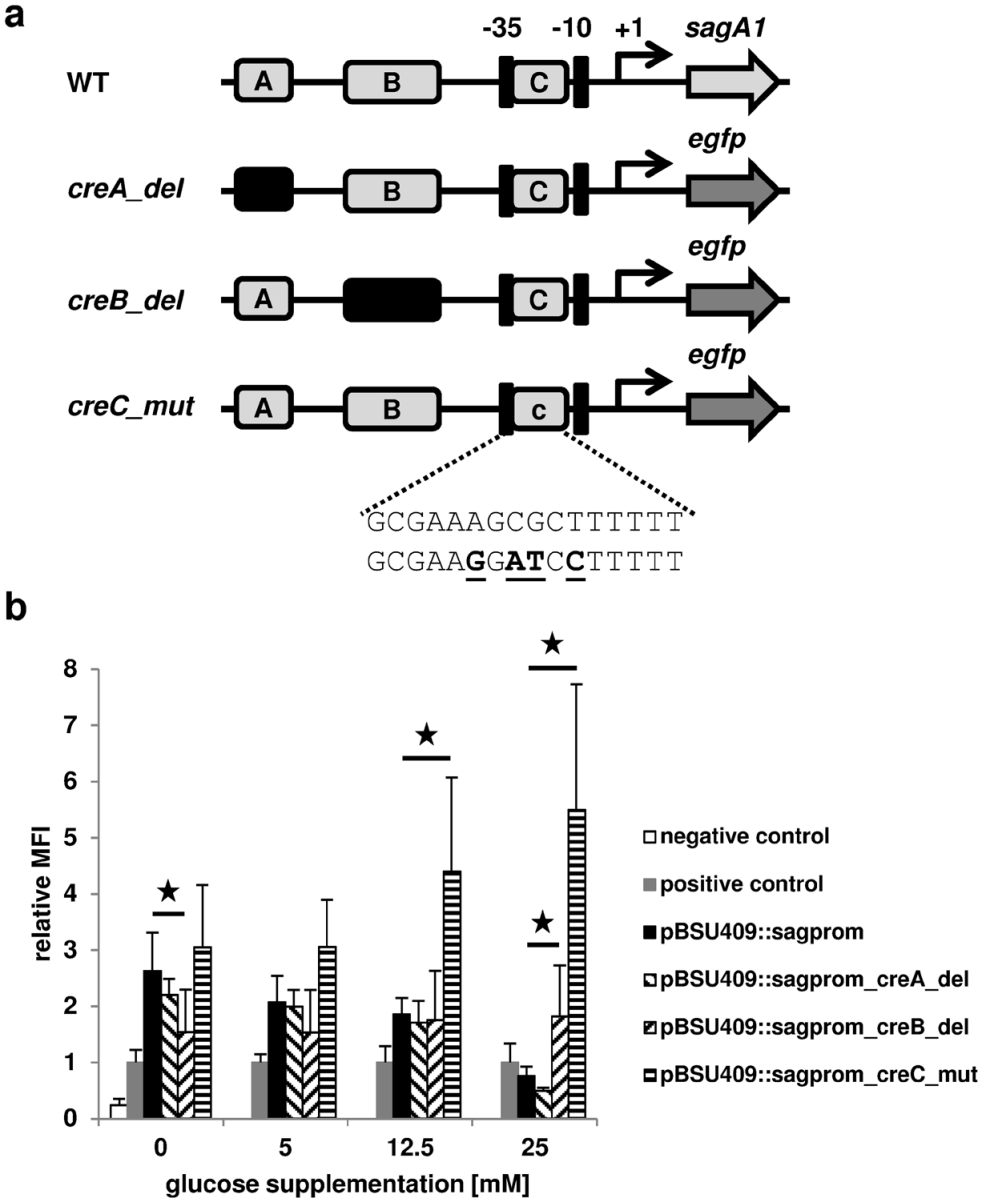
(**a**) Schematic representation of reporter plasmids with mutated SLS promoters used to investigate the *in silico* predicted *cre* sites. WT: promoter of SLS; *creA_del*: pBSU409*::sagprom_creA_del* (pBSU881); *creB_del*: pBSU409*::sagprom_creB_del* (pBSU880); *creC_mut*: pBSU409*::sagprom_creC_mut* (pBSU876). (**c**) The sequence of the predicted *cre* site (upper row) and the mutations of the sequence (bold and underlined) in pBSU409*::sagprom_creC_mut* (lower row) are shown. (**b**) Effect of glucose supplementation on the activity of mutated SLS promoters in the *S*. *anginosus* type strain. The relative mean fluorescence intensity of cells grown in THY medium supplemented with the indicated glucose concentrations is shown in comparison to the positive control. Positive control: *S*. *anginosus* pBSU409*::cfbprom*; Negative control: *S*. *anginosus* pBSU409. The mean values and standard deviations of five independent experiments are shown. Mann-Whitney-U test was performed to illustrate significant difference to *S*. *anginosus* pBSU409*::sagprom* (p < 0.05).

To investigate the influence of the *in silico* predicted *cre* sites on the observed glucose dependent repression of the SLS promoter activity, we mutated the putative *cre* sites in the reporter plasmid pBSU409*::sagprom*. The sequences of *creA* and *creB* were separately deleted and the altered promoters were cloned in front of *egfp* in the reporter plasmid (Fig. 4a). Since the *creC* site overlaps the bacterial promoter, we could not create a deletion of this sequence. Instead we mutated four putative important nucleotides of *creC* that should diminish the binding of CcpA to this site. All of the three different reporter plasmids carrying the mutated SLS promoters were transformed separately into the *S*. *anginosus* type strain and the promoter activity was determined for cells grown in medium with increasing glucose supplementations (Fig. 4b). The strain carrying the reporter plasmid with the *creA* deletion showed a similar behavior like the strain containing the wild type promoter with a concentration dependent reduction of promoter activity. Deletion of *creB* in the promoter results in a lower promoter activity compared to the wild type promoter without glucose supplementation and showed constant expression of *egfp* without significant differences for varying glucose concentrations. Compared to the wild type promoter, the promoter activity with 25 mM glucose supplementation was significantly higher in the *creB* deletion. In contrast to the previous findings the mutation of *creC* completely abolishes the glucose dependent promoter repression. This strain shows a constantly high SLS promoter activity with increasing glucose concentrations in the growth medium most closely resembling the expression data obtained for the *S*. *anginosus ΔccpA* strain.

### CcpA binds to *creC* site *in vitro*

**Figure 5:**
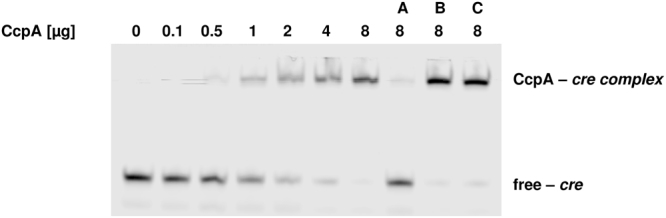
EMSA of *creC* using His-tagged CcpA. Increasing amounts of purified CcpA (0–8 µg) were used and assayed for binding to labeled *creC* (GTTTAC**GCGAAAGCGCTTTTTT**TATATA). The specificity of binding was assayed using 500-fold molar excess of unlabeled *creC* (A), *creC_mut* (B GTTTAC**GCGAAGGATCCTTTTT**TATATA) and *creB* (C; TGCTAT**AAGAACGCGCTTTTTATTTTGTTTTA**GATGGT).

The data obtained in the reporter plasmid assay prompted us to further characterize the interaction of CcpA with the potential *cre* binding sites. To investigate if CcpA is able to directly regulate the SLS expression by binding to the *in silico* predicted *cre* sequences, we performed electrophoretic mobility shift assays (EMSA) with purified His-tagged CcpA. Several studies reported that CcpA is able to bind to *cre* sites in the absence of HPr and the allosteric effector fructose-1,6-bisphosphate^23,30,32,33^. Therefore we performed the EMSA with CcpA alone and a promoter fragment harboring *creC* (GTTTAC**GCGAAAGCGCTTTTTT**TATATA). Increasing amounts of CcpA (0.5–8 µg) induced a shift of labeled *creC*, indicating binding of CcpA to the DNA (Fig. 5). The addition of a 500-fold molar excess of unlabeled *creC* was able to inhibit the observed shift (Fig. 5, lane A) whereas the addition of the same amount of unlabeled mutated *creC* (GTTTAC**GCGAAGGATCCTTTTT**TATATA) and unlabeled *creB* had no effect (Fig. 5, lane B and C). Thus, we were able to demonstrate that CcpA binds to the *creC* site located in the promoter of the SLS operon.

## Discussion

Bacteria sense the environmental conditions in order to tightly regulate their gene expression. One global regulatory mechanism that bacteria exert to conserve energy is CCR. The bacteria thereby regulate the uptake and consumption of different carbohydrates by downmodulation of alternative sugar utilization pathways in the presence of preferred substrates. This CCR mechanism was also demonstrated to play an important role in disease progression and virulence gene expression in different Gram-positive pathogens. In *Staphylococcus aureus* CcpA affects the expression of important virulence factors and it is required for pathogenesis^34,35^. The enolase and suilysin expression is regulated by CcpA in *Streptococcus suis* and a *ccpA* mutant was attenuated in a murine infection model^36^. Additionally, the capsule biosynthesis is affected by CcpA in different pathogens^31,34,37^ and CcpA was demonstrated to regulate the SLS expression in *S*. *pyogenes* by binding to a *cre* site located in the SLS promoter^24^.

Despite the increased knowledge about the epidemiology of *S*. *anginosus*^1–6^, the pathogenicity mechanisms in this species are poorly investigated^38^. One exception is the SLS responsible for the β-hemolytic phenotype of the *S*. *anginosus* type strain. The SLS of *S*. *anginosus* is a broad-range hemolysin able to lyse erythrocytes of different origins. The activity of the hemolysin is temperature dependent and a reduced SLS expression was demonstrated in cells growing in the presence of high glucose concentrations^11,14^. Such an expression pattern indicates a CCR mechanism controlling the SLS expression. We therefore constructed a *ccpA* deletion mutant and investigated the activity of the SLS promoter using a GFP reporter system. The observed reductions of the promoter activity in the *S*. *anginosus* wild type strain is absent in the *ccpA* mutant indicating that the SLS operon is controlled by CcpA. Even without glucose supplementation, the mutant strain showed an increased promoter activity which could be explained by the THY growth medium that already contains 11 mM glucose.

To investigate the potential effect of the reduced hemolysin expression in the presence of high glucose concentrations on the hemolytic activity of *S*. *anginosus* cells we performed hemolysis assays with human erythrocytes. The wild type as well as the *∆ccpA* strain showed hemolytic activity up to 12.5 mM glucose supplementation. The observed reduction of the promoter activity measured in the wild type strain under these conditions thereby seemed to be sufficient for complete lysis of the erythrocytes. The wild type cells growing with 25 mM glucose supplementation showed no hemolytic activity at all whereas the *∆ccpA* strain was still hemolytic. The complemented *S*. *anginosus ∆ccpA::ccpA* showed the same behavior like the wild type strain, thus confirming the role of CcpA in the control of hemolysis.

To investigate if CcpA directly or indirectly regulates the hemolysin expression, we screened the SLS promoter for potential *cre* sites. To reduce the possibility of missing a putative *cre* site, we allowed three mismatches to the already degenerate consensus *cre* sites and we included the published *cre2* site in our analysis although this site was so far only reported in *S*. *suis*^23^. The *in silico* prediction identified three putative *cre* sites in the SLS promoter including the *creC* site that overlaps the −35 region of the bacterial promoter^10^. The location of the *cre* site in respect to the transcriptional start site is determining the effect of CcpA on the transcription and a position overlapping the bacterial promoter would indicate that CcpA is a strong repressor of the indicated gene^39^. To investigate the influence of the *in silico* predicted *cre* sites on the observed glucose dependent repression of the SLS transcription, we performed the promoter reporter assay with plasmids containing mutated SLS promoters. The deletion of the *creA* site resulted in an expression pattern like the wild type promoter demonstrating that this site is no CcpA binding site. For the *creB* deletion a low but constant reporter expression was measured at all tested glucose concentrations. As the *creC* site overlaps the −35 region of the bacterial promoter we mutated potentially conserved nucleotides of its sequence instead of deleting the *cre* site as this would probably destroy the promoter. The expression pattern measured with the *creC* site mutation resembles the pattern observed in the *∆ccpA* strain, with a trend towards increased expression with higher glucose concentrations which is not significant. Thus, indicating that the *creC* site is most relevant for the glucose dependent reduction of the SLS transcription.

To verify that CcpA directly regulates SLS expression by binding to the *creC* site, we performed band shift experiments using His-tagged CcpA alone as previous studies reported that CcpA is able to bind to *cre* sites in the absence of serine phosphorylated HPr^23,30,32,33^. Increasing amounts of CcpA were able to shift the labeled *creC* site. Excess amounts of unlabeled *creC* were able to compete for binding of CcpA to the labeled *creC*. The specificity of binding was tested with an unlabeled mutated *creC* sequence. CcpA was not able to bind to this site suggesting that the interaction of CcpA and *creC* is specific. The presence of more than one *cre* site in the promoter of a CcpA regulated gene was already described^23,39^. We observed in our promoter reporter assay that the putative *creB* site deletion resulted in an increased promoter activity with 25 mM glucose supplementation compared to the wild type. To analyze the potential binding of CcpA to this sequence we used the *creB* sequence as specific competitor in the band shift experiment but this sequence was not able to compete for CcpA binding to the *creC* motif. This indicates that under the conditions tested CcpA does not bind to *creB*. However, it cannot be ruled out that the *creB* site is involved in the regulation of the SLS expression *in vivo* as it was reported that serine phosphorylated HPr increases the affinity of CcpA to *cre* sites^40,41^.

In conclusion, we report the first investigation of the regulation of a potential virulence factor in *S*. *anginosus* and we demonstrate that CcpA directly regulates the SLS expression of *S*. *anginosus* by binding to a *cre* site overlapping the bacterial promoter.

## Materials and Methods

### Bacterial strains and growth conditions

The *S*. *anginosus* and *Escherichia coli* strains used in this study are summarized in Table 1. The *S*. *anginosus* type strain was incubated on Sheep blood agar plates (TSA + SB, Oxoid, Basingstoke, UK) at 37 °C and 5% CO_2_ atmosphere. Liquid cultures of *S*. *anginosus* were incubated in THY broth (Todd-Hewitt Broth [Oxoid] supplemented with 0.5% yeast extract [BD, Miami, USA]) and supplemented with appropriate antibiotics if necessary. *S*. *anginosus* strains carrying pAT28 derivatives were grown on THY agar plates supplemented with 120 µg ml^−1^ spectinomycin and *S*. *anginosus* strains containing pG^+^host5 vector were incubated on TSA + SB in the presence of 5 µg ml^−1^ erythromycin. *E*. *coli* strains were routinely incubated aerobically in LB medium at 37 °C and appropriate antibiotics were added if necessary. The *E*. *coli* DH5α strain served as cloning host for pAT28 plasmids and *E*. *coli* EC101 was used for pG^+^host5 vectors with 100 µg ml^−1^ spectinomycin and 400 µg ml^−1^ erythromycin respectively. *E*. *coli* BL21(DE3) containing a pET21a derivative was grown in presence of 100 µg ml^−1^ ampicillin.

**Table 1:** Strains and plasmids.

Strain or plasmid	Definition	Source
***S***. ***anginosus***		
BSU 458	*S*. *anginosus* type strain ATCC33397, Hly+	ATCC
BSU 554	BSU 458 derivative, carrying pBSU409	^44^
BSU 556	BSU 458 derivative, carrying pBSU409*::cfbprom*	^45^
BSU 805	BSU 458 derivative, carrying pBSU409*::sagprom*	^11^
BSU 926	BSU 458 derivative; *∆ccpA*	This study
BSU 928	BSU 458 derivative; *∆ccpA*, carrying pBSU409*::sagprom*	This study
BSU 948	BSU 928 derivative; *∆ccpA::ccpA*	This study
BSU 886	BSU 458 derivative; carrying pBSU409*::sagprom_creA_del*	This study
BSU 913	BSU 458 derivative; carrying pBSU409*::sagprom_creB_del*	This study
BSU 916	BSU 458 derivative; carrying pBSU409*::sagprom_creC_mut*	This study
***E***. ***coli***		
EC101	*E*. *coli* JM101 derivative with *repA* from pWV01 integrated into the chromosome	^46^
DH5α	*endA1 hsdR17 supE44* DlacU169(f80lacZDM15) *recA1 gyrA96 thi-1 relA1*	Boehringer
BL21(DE3)	*E*. *coli* BL21 derivative with DE3 λ prophage carrying the T7 RNA polymerase gene and *lacI*^q^	Novagen
BSU 957	BL21 derivative, carrying pET21a-*ccpA*-N-His	This study
pAT18	*lacZα*, ori pUC, ori pAmβ1, Em^R^	^47^
pAT28	*lacZα*, ori pUC, ori pAmβ1, Spc^R^	^48^
pBSU409	pAT28 derivative carrying *egfp*	^44^
pBSU803	pBSU409*::sagprom*; pBSU409 derivative, *egfp* under the control of the SLS promoter	^11^
pBSU881	pBSU409*::sagprom_creA_del*; pBSU803 derivative carrying a deletion of *creA*	This study
pBSU880	pBSU409*::sagprom_creB_del*; pBSU803 derivative carrying a deletion of *creB*	This study
pBSU876	pBSU409*::sagprom_creC_mut*; pBSU803 derivative carrying mutations in *creC*	This study
pET21a	*lacI*, ori F1, ori pBR322, T7 promoter and terminator, Amp^R^	Novagen
pET21a-*ccpA*-N-His	pET21a derivative carrying *ccpA*	This study
pG^+^host5-*ccpA∆100nt*	pG^+^host5 derivative carrying up- and downstream sequences of *ccpA*	This study

### General DNA techniques

Genomic DNA (GenEluteTM Bacterial Genomic DNA Kits, Sigma-Aldrich, St. Louis, MO) and plasmid DNA (QIAprep® Spin Miniprep Kit, Qiagen, Hilden, Germany) was isolated following standard procedures of the manufacturers. Taq polymerase (Roche, Mannheim, Germany) was used for Polymerase Chain Reactions (PCR) with an initial denaturation step of 3 min 94 °C, 30 amplification cycles of 1 min 94 °C, 30 sec. 50 °C, 1–4 min 72 °C and a final elongation of 7 min 72 °C. Oligonucleotides used in this study are summarized in Table 2.

**Table 2:** Primers used in this study.

Name	Sequence	No.
ccpA_pGh_F1_fwd_SalI	CCGATTGTCGACGGTCATTCTAGTACCTTC	1
ccpA_pGh_F1_rev	CAGAAACTTCCTTGTTATGGAATGACAACTCCAACAGTC	2
ccpA_pGh_F2_fwd	TAACAAGGAAGTTTCTGTTG	3
ccpA_pGh_F2_rev_EcoRI	GGCGGCGAATTCTACCTGCGAATCATC	4
ccpA_integration_rev	CCGAGACACTAGTATCTCAG	5
ccpA_integration_fwd	TCAAGAGGACAGTAAGAAC	6
ccpA_comp_F1_rev	GGGCGGTAATCCAAGCGGTC	7
ccpA_comp_F2_fwd	CTTGGATTACCGCCCAAATG	8
ccpA_comp_F2_rev	GTTTGCTTCTAAGCCACAAAGGTATAGAGCC	9
ccpA_comp_F3_fwd	CTTAGAAGCAAACTTAAGAGTGTG	10
ccpA_comp_F3_rev	CGATACAAATTCCCCGTAGGC	11
ccpA_comp_F4_fwd_2	CGGGGAATTTGTATCGGGGTATAATAGAAGAC	12
ccpA_comp_F4_rev	GTCCCGCACAGACAACCAC	13
sagprom_fwd	GGGCCCGAATTCGGTTGGATTTGATAGTAATGTACG	14
sagprom_rev	GGGCCCGGATCCGAAGAAAATTTTAACATAGTTTG	15
cre2_F1_rev(creA)	CCTTTGTCATGTTTTATTCACTCAGATGATAATAATTCTG	16
cre2_F2_fwd	GTGAATAAAACATGACAAAG	17
cre1_F1_rev(creB)	CACAAATATAACCATCATAGCATTTGAACACAC	18
cre1_F2_fwd	GATGGTTATATTTGTGAAATAGG	19
cre3_mut_fwd(creC)	CGCGAAGGATCCTTTTTTATATAATGTG	20
cre3_mut_rev	TATATAAAAAAGGATCCTTCGCGTAAAC	21
ccpA_N_His6_fwd	GAAGACATATGCACCACCACCACCACCACAACACAGACGATACAGTAACC	22
ccpA_N_His6_rev	GTCGGCCTCGAGTTACTTTCTTGTTGAACG	23
IRD700_cre_fwd(creC)	IRDye700-GTTTACGCGAAAGCGCTTTTTTTATATA	24
cre_rev(creC)	TATATAAAAAAAGCGCTTTCGCGTAAAC	25
cre_mut_fwd(creC_mut)	GTTTACGCGAAGGATCCTTTTTTATATA	26
cre_mut_rev(creC_mut)	TATATAAAAAAGGATCCTTCGCGTAAAC	27
creB_fwd	TGCTATAAGAACGCGCTTTTTATTTTGTTTTAGATGGT	28
creB_rev	ACCATCTAAAACAAAATAAAAAGCGCGTTCTTATAGCA	29

### Construction of *ccpA* deletion and complementation strain

The temperature sensitive pG^+^host5 vector was used for the creation of the *S*. *anginosus ∆ccpA* strain^42^. Briefly, the up- and downstream regions of the target were amplified using Primers 1/2 and 3/4. The resulting PCR products were fused in an overlap-extension PCR (OE-PCR) with Primer 1/4 and were subsequently cloned into the pG^+^host5 plasmid. The resulting deletion vector pG^+^host5-*ccpA∆100nt* was transformed into the *S*. *anginosus* type strain using the competence stimulating peptide CSP-1 as described previously^43^ and the regeneration of the transformants was performed for 3 h at 39 °C to directly obtain integration mutants. Cells were plated on TSA + SB plates supplemented with 5 µg ml^−1^ erythromycin and incubated at 37 °C for two days. Erythromycin resistant clones were tested via PCR for the integration of the plasmid at the desired locus. To induce loss of the chromosomal integration of pG^+^host5, cultures were grown for 8 h at 30 °C. Subsequently single clones were tested for erythromycin susceptibility and the generation of the desired 100 bp deletion was verified by sequencing.

The *S*. *anginosus ∆ccpA::ccpA* strain was constructed by a chromosomal integration of the *ccpA* gene at its native locus. A linear DNA fragment consisting of the *ccpA* gene and an erythromycin resistance gene flanked by the up- and downstream sequence of *ccpA* was constructed via OE-PCR. First, a silent point mutation was introduced into the *ccpA* gene to be able to distinguish the complemented strain and the type strain. Therefore two PCR products (F1: Primer 6/7, F2: Primer 8/9) were fused in an OE-PCR using Primer 6/9. The erythromycin resistance gene was amplified with Primer 10/11 and pAT18 as template and fused to the OE-PCR product with Primers 6/11. In a final OE-PCR the 500 bp downstream region of *ccpA* (Primer 12/13) was fused to the previously constructed DNA using Primer 6/13 generating the final *ccpA* complementation fragment. The linear PCR product was transformed into *S*. *anginosus ∆ccpA* using CSP-1 and the integration of the PCR product in the *ccpA* locus was verified by PCR and sequencing leading to the creation of *S*. *anginosus ∆ccpA::ccpA*.

### Promoter reporter assay

To investigate the SLS promoter activity an EGFP reporter system was used as previously described^11^. Briefly, *S*. *anginosus* carrying pBSU409 derivatives were grown in THY medium with glucose supplementations of 0, 5, 12.5 and 25 mM for 16 h at 37 °C and the expression of EGFP was measured using fluorescence-activated cell sorting (FACSCalibur; Becton Dickinson Immunocytometry Systems, San Jose, CA). To determine the influence of CcpA on the SLS promoter activity the pBSU409*::sagprom* plasmid was transformed in *S*. *anginosus ∆ccpA*. For the characterization of potential *cre* sites mutated SLS promoters were constructed and cloned in front of *egfp* in pBSU409. The *creA* deletion was generated by OE-PCR. In a first step two PCR fragments were amplified using Primer 14/16 and 17/15 which were afterwards fused with Primer 14/15. The resulting SLS promoter with the *creA* deletion was cloned in pBSU409 leading to the formation of plasmid pBSU409*::sagprom_creA_del*. The same procedure was used for the *creB* deletion (Primer 14/18 and 19/15) and the *creC* mutation (Primer 14/20 and 21/15) leading to pBSU409*::sagprom_creB_del* and pBSU409*::sagprom_creC_mut*. The plasmids were transformed into the *S*. *anginosus* type strain and the SLS promoter activity was determined. The *S*. *anginosus* pBSU409 strain served as negative control^44^ and the *S*. *anginosus* pBSU409*::cfbprom* strain carrying the CAMP-factor promoter (*cfb*) of *S*. *agalactiae* was used as positive control^45^. Depicted are the mean fluorescence intensities of the different strains normalized to the values obtained for the positive control in the presence of indicated glucose concentration.

### Hemolysis assay

The hemolysis assay was carried out with human erythrocytes. Peripheral blood was collected from healthy adult volunteers who gave written informed consent to donate blood for the use in the study. The ethics committee at the University of Ulm approved this procedure (Certificate No. 23/13) and the methods were carried out in accordance with the regulations. The hemolytic activity of the *S*. *anginosus* type strain, the *S*. *anginosus ∆ccpA* and the *S*. *anginosus ∆ccpA::ccpA* strain was measured as described previously with some modifications^11^. The cells were grown overnight in THY medium with glucose supplementations of 0, 5, 12.5 and 25 mM and treated as previously described. A serial dilution of the cells was generated and 100 µl of each dilution was incubated together with 100 µl of a 0.5% erythrocyte solution at 37 °C for 1 h in a 96-well plate with conical bottom. After centrifugation, 100 µl of the supernatant was transferred to a 96-well plate with flat bottom and the Absorption at 540 nm corresponding to free hemoglobin was measured in a plate reader. As positive control served ddH_2_O and PBS was used as negative control.

### Expression and purification of His-tagged CcpA

The CcpA protein of *S*. *anginosus* was expressed using the pET21a system and *E*. *coli* BL21(DE3). For the generation of the expression plasmid, *ccpA* was amplified (Primer 22/23) and cloned into pET21a leading to the formation of pET21a-*ccpA*-N-His. Expression of recombinant N-terminal His-tagged CcpA was induced in mid-logarithmic growth of *E*. *coli* BL21 using 0.1 mM isopropyl-β-D-thiogalactopyranoside. Cells were harvested by centrifugation after 8 h incubation at 30 °C and bacterial pellets were stored at −20 °C. CcpA was purified under native conditions using the Protino^®^ Ni-TED 1000 Packed Columns Kit (Macherey-Nagel GmbH & Co. KG, Düren, Germany) following the instructions of the manufacturer. After purification, the elution buffer was exchanged to TKED buffer (100 mM Tris-HCl, 150 mM KCl, 1 mM EDTA, 0.1 mM dithiothreitol)^32^ using Amicon® Ultra Centrifugal filters (30 K, Merck Millipore Ltd, Carrigtwohill, Ireland) and the protein concentration was determined using the Quick Start^TM^ Bradford Protein Assay (Bio-Rad Laboratories, Hercules, CA).

### Electrophoretic mobility shift assay (EMSA)

Double stranded DNA probes were constructed by annealing sense and antisense oligonucleotide 24/25 (*creC*), 26/27 (*creC_mut*) and 28/29 (*creB*). Briefly, equal molar amounts of sense and antisense oligonucleotide were mixed in Annealing Buffer (10 mM Tris-HCl, 1 mM EDTA, 50 mM NaCl, pH 8.0) and annealing was performed in a PCR machine (5 min 95 °C, 70 cycles of 1 min reducing the temperature by 1 °C per cycle). Oligonucleotides were purchased from metabion international AG (Planegg, Germany) with oligonucleotide 24 (IRD700_cre_fwd) labeled with the fluorescent dye IRDye700 at the 5′ end. EMSA was performed with a constant amount of labeled oligonucleotide probe (5 pmol) and increasing amounts of CcpA (0–8 µg). Each reaction was incubated for 30 min at room temperature in EMSA binding buffer (20 mM Tris-HCl, 1.5 mM dithiothreitol, 1 mM EDTA, 75 mM NaCl, 1 µg poly(dI-dC), 5% glycerol, pH 8.0). The DNA-protein complexes were separated on native 6% polyacrylamide gels in 0.5 × TBE buffer and were analyzed using the Odyssey Clx near-infrared fluorescence imaging system (LI-COR Corporate Offices, Lincoln, NE).

### Equipment and Settings

Analysis of EMSA pictures was performed using the Image Studio Version 5.2 (LI-COR Corporate Offices, Lincoln, NE). The 700 nm channel was used in the DNA Gel Setup with a Scan resolution of 42 µm and a Focus Offset of 2.0 mm.

### Bioinformatical and statistical analysis

Nucleotide sequences were retrieved from the GenBank database (http://www.ncbi.nlm.nih.gov/). For statistical analysis the nonparametric Mann-Whitney U test was performed and datasets were considered significant with a p-value smaller than 0.05.

### Data availability

The datasets generated during and/or analyzed during the current study are available from the corresponding author on reasonable request.
